# ANFIS Fuzzy convolutional neural network model for leaf disease detection

**DOI:** 10.3389/fpls.2024.1465960

**Published:** 2024-11-05

**Authors:** Tae-hoon Kim, Mobeen Shahroz, Bayan Alabdullah, Nisreen Innab, Jamel Baili, Muhammad Umer, Fiaz Majeed, Imran Ashraf

**Affiliations:** ^1^ School of Information and Electronic Engineering and Zhejiang Key Laboratory of Biomedical Intelligent Computing Technology, Zhejiang University of Science and Technology, Hangzhou, Zhejiang, China; ^2^ Department of Artificial Intelligence, The Islamia University of Bahawalpur, Bahawalpur, Punjab, Pakistan; ^3^ Department of Information Systems, College of Computer and Information Sciences, Princess Nourah bint Abdulrahman University, Riyadh, Saudi Arabia; ^4^ Department of Computer Science and Information Systems, College of Applied Sciences, AlMaarefa University, Riyadh, Saudi Arabia; ^5^ Department of Computer Engineering, College of Computer Science, King Khalid University, Abha, Saudi Arabia; ^6^ Department of Computer Science and Information Technology, The Islamia University of Bahawalpur, Bahawalpur, Pakistan; ^7^ Department of Software Engineering, University of Gujrat, Gujrat, Pakistan; ^8^ Department of Information and Communication Engineering, Yeungnam University, Gyeongsan, Republic of Korea

**Keywords:** plant disease detection, deep neural networks, ANFIS, image processing, deep learning

## Abstract

Leaf disease detection is critical in agriculture, as it directly impacts crop health, yield, and quality. Early and accurate detection of leaf diseases can prevent the spread of infections, reduce the need for chemical treatments, and minimize crop losses. This not only ensures food security but also supports sustainable farming practices. Effective leaf disease detection systems empower farmers with the knowledge to take timely actions, leading to healthier crops and more efficient resource management. In an era of increasing global food demand and environmental challenges, advanced leaf disease detection technologies are indispensable for modern agriculture. This study presents an innovative approach for detecting pepper bell leaf disease using an ANFIS Fuzzy convolutional neural network (CNN) integrated with local binary pattern (LBP) features. Experiments involve using the models without LBP, as well as, with LBP features. For both sets of experiments, the proposed ANFIS CNN model performs superbly. It shows an accuracy score of 0.8478 without using LBP features while its precision, recall, and F1 scores are 0.8959, 0.9045, and 0.8953, respectively. Incorporating LBP features, the proposed model achieved exceptional performance, with accuracy, precision, recall, and an F1 score of higher than 99%. Comprehensive comparisons with state-of-the-art techniques further highlight the superiority of the proposed method. Additionally, cross-validation was applied to ensure the robustness and reliability of the results. This approach demonstrates a significant advancement in agricultural disease detection, promising enhanced accuracy and efficiency in real-world applications.

## 1 Introduction

Plant leaf diseases pose a significant threat to global agriculture, affecting crop yields and quality. Common diseases include fungal infections (e.g., powdery mildew, rust), bacterial diseases (e.g., bacterial blight), and viral infections (e.g., mosaic virus). Early and accurate detection of these diseases is crucial for effective management and to minimize economic losses. Historically, disease detection relied on manual inspection by farmers or experts, which is labor-intensive and prone to human error. This approach also limits the ability to scale disease monitoring over large areas. Traditional laboratory methods, such as microscopy and culture tests, offer more accuracy but are time-consuming and require specialized equipment. Recent advancements in remote sensing and machine learning (ML) have revolutionized plant disease detection. Technologies such as hyperspectral imaging, drones, and satellite imagery enable the non-invasive monitoring of crops over large areas. These tools capture data on various spectral bands, which can be analyzed to identify stress or disease symptoms that are not visible to the naked eye. The pathogens are found to be the causal agents of these diseases and infections, which comprise parasitic plants, viruses and viroids, bacteria, and fungi. Pathogens are infectious organisms leading to plant diseases. Aside from this, there are other plant-feeding organisms such as vermin, insects, mites, and other microbes that aggravate plant health issues. Some bacteria cause diseases that injure plants, however, the majority of bacteria are benign and saprotrophic Das et al. (2020). An illustration of known bacteria that cause diseases in plants are Phytoplasmas and Spiroplasmas.

Agriculture is the backbone of the world’s economy, not only because it is the primary source of food only, but also because of industrial raw materials Tichkule and Gawali (2016); Jamil et al. (2022). Agriculture and planting are expedient factors in our survival, as they are used to provide oxygen and food. Simultaneously, practical approaches have been implemented in better production of crops and increasing their fighting capacity against diseases and pests. Diseases that infect plants affect all animals, relying on plants in a variety of ways, either directly or indirectly Bharali et al. (2019). Any portion of the plant, including the roots, stems, branches, and leaves, can be impacted by plant diseases. Additionally, different causative organisms cause different plant diseases, some are caused by bacteria, others by fungi, and others by viruses Husin et al. (2012). Climate change favors the spread of diseases in crops. Crop diseases are misidentified, hence negatively affecting the yield of such crops. Plant diseases can be divided into two significant groups: abiotic and biotic Balakrishna and Moparthi (2020). The former, abiotic diseases, are generally brought about by non-living aspects of an environment, for example, meteorological factors such as weather, temperature, humidity, and some specific chemicals. On the other hand, the latter is caused by living elements of an ecosystem, for example, fungi, bacteria, viruses, and other organisms. Point spread of various pathological diseases and pests, including invasive ones, is among the most disastrous issues of modern agriculture Elad and Pertot (2014); Meng et al. (2023). Concerning these issues, it is reasonably required to monitor plant diseases and pests promptly. Techniques for remote sensing have a lot of promise for solving these problems Mahlein et al. (2018).

This indicates that passive and active remote sensing technologies are the two types. The latter includes LiDAR and radar, but the former includes only the so-called optical Kim et al. (2024, Kim et al., 2021). Depending on the sensors’ spectral resolution, two categories of passive optical remote sensing can typically be distinguished: multispectral and hyperspectral Jensen (2006). Among passive remote sensing methods, hyperspectral sensing has vast potential as a device that measures reflected sun radiation to track biotic and abiotic plant stress in a non-invasive, non-destructive manner Jones and Vaughan (2010). This is a technique for collecting and storing information from an object’s spectroscopy in a third-dimensional spectral cube containing hundreds of consecutive wavelengths and spatial data. Hyperspectral imaging widely allows for the opportunities to detect an early sign of the disease by providing preliminary indicators in the form of minute variations in spectrum reflectance brought on by reflection or absorption. Since hyperspectral photos provide a very comprehensive spectral profile thanks to their hundreds of spectral bands, they are highly useful for identifying even the smallest differences in soil, canopies, or even leaves. In this sense, hyperspectral imaging may be applied to new problems about the precise and prompt assessment of crops’ physiological status. It may help in early detection of disease spread and pest incidence, which might prevent heavy crop loss, reduce usage of pesticides, and minimize negative impacts on the environment and human health, hence improving the current status of integrated pest management (IPM) practices Lucieer et al. (2014); Gonzalez-Dugo et al. (2015).

Recently, a variety of small hyperspectral sensors have been created for usage in commercial settings, including FireflEYE, VNIR HySpex, and Micro-Nano-Hyperspec Adao et al. (2017). The above-listed sensors could be installed on manned and unmanned aerial vehicles (UAVs), helicopters, and airplanes, among other platforms, to obtain hyperspectral imaging for a range of monitoring tasks Metternicht (2003); Schell and Deering (1973). There are several varieties of hyperspectral cameras, such as whisk-broom, push-broom, and snapshot cameras. Currently, the use of mobile phone cameras is being widely utilized to capture earth observation data.

Accurate and timely identification of plant diseases represents a significant challenge to agricultural experts and farmers. Recent changes in digital image processing present a practical and effective solution for diagnosing and classifying disease symptoms for better yield Kumar and Raghavendra (2019); Islam et al. (2024). Artificial methods with remote sensing data may soon help increase crop yields with the early identification of diseases affecting plant leaves, curtailing the entrance of disease to other nearby crops. Even at an early stage, computerized image processing techniques can identify the disease and reduce its potential to affect the entire crop Devaraj et al. (2019). In the long term, the proposed work will help establish timely disease detection thereby improving crop yield and food supply. The PlantVillage dataset, a common collection of leaf photos taken in fields, served as the study’s dataset Rajasekaran et al. (2020); Zhang et al. (2023). The primary objective is to develop a system that uses Earth observation data to categorize leaves as healthy or unhealthy automatically. The contributions to be achieved in this study can be summarized as follows:

- This research proposes a novel framework that uses local binary pattern features (LBP) with ANFIS Fuzzy customized convolutional neural network (called PlantNet) for giving a high accuracy rate in the classification between healthy and bacterial-infected using plant leaf images of pepper bell.
- LBP features greatly reduce the computational complexity overhead while ANFIS Fuzzy customized CNN performs well for the infected regions portion detection and classification.
- To evaluate the performance of PlantNet, the Plant Village benchmark dataset is utilized and performance is compared with four ML and four deep learning (DL) models.
- The PlantNet performance is precisely compared with previously published research works that successfully affirm the superiority of PlantNet. The results of PlantNet are further generalized using cross-validation techniques.

This research is structured as follows: Section 2 discusses previously published studies carried out in this field, and Section 3 outlines the suggested methodology. It also contains preprocessing procedures and a model description. The evaluation of the suggested approach is the focus of Section 4, which is followed by a discussion of experimental findings. The conclusion is given in Section 5.

## 2 Related work

This section offers an overview of many cutting-edge approaches that have been utilized in the past to identify pepper bell plant leaf diseases. In previous years, pre-processing methods have been used on several plant leaf photos to accurately identify the various plant diseases. Most of the studies mainly utilized refining and polishing image processing filters to improve the picture quality. Other filters have also been adopted to remove additive noise in these images. A deep CNN powered by Bayesian learning was proposed by Sachdeva et al. (2021) as a means of automatically identifying plant leaf diseases. Images of potato, tomato, and pepper bell leaves were used in their investigation. With 98.9% accuracy, the prototype design featured several hierarchical tiers cooperating inside a Bayesian framework. To categorize pepper leaf illnesses, In this line, the authors proposed a transfer learning (TL) model Zeng et al. (2021). They solely employed the MobileNetV2 model in this instance. According to the experimental findings, the accuracy of the MobileNetV2-based TL model was 99.55%.

The study by Devi and Amarendra (2021) developed an automatic classification approach for bell pepper plant leaf diseases using ML. The HGB algorithm with multiple feature extraction techniques was proposed by the authors, who also employed several ML models. According to their findings, bell pepper leaf illnesses could be classified with an output of 89.11% using the combination of HGB classification and the features of the images’ LBP and fused histogram of oriented gradients (HoG). When Mohanty et al. (2016) tested their TL models to identify leaf diseases in a variety of plants, they found that the GoogLeNet and AlexNet TL models had train-test split model classification accuracy of 80:20 and 70:30, respectively. In comparison, the current study achieved a 99.34% validation accuracy with the GoogLeNet 80:20 train-test split model.

Smith et al. (2023) discusses precision agriculture technologies that leverage remote sensing and TL techniques. The authors highlight the significant advantages of integrating TL with remote sensing data to improve crop monitoring, yield prediction, and disease detection. By utilizing pre-trained models on large datasets and fine-tuning them with agricultural-specific data, the studies reviewed demonstrate enhanced performance in various agricultural tasks. The authors also discuss the limitations of TL in this domain, such as the requirement of high-quality labeled datasets and the potential for overfitting. Overall, the review provides valuable insights into the advancements and challenges in the application of TL and remote sensing in precision agriculture. In a review paper by Johnson et al. (2023), the authors focus on the applications of DL and TL in remote sensing for crop management in precision agriculture. The authors explore various DL architectures, such as convolutional neural networks (CNNs) and RNNs, which have been successfully applied to analyze remote sensing data for tasks like crop classification, disease detection, and soil moisture estimation. The use of TL in these applications is particularly highlighted as a means to reduce training time and improve model accuracy by leveraging knowledge from models pre-trained on non-agricultural datasets. The paper also addresses the challenges of implementing these techniques, including the need for large and diverse datasets, and the potential biases introduced by TL from non-agricultural domains.

The integration of remote sensing and TL techniques to enhance precision agriculture was examined in research by Chen et al. (2024); Williams et al. (2024). They discuss various case studies where remote sensing data, combined with TL approaches, have been used to improve the accuracy of agricultural applications such as weed detection, yield estimation, and crop health monitoring. The authors highlight the benefits of using TL to adapt models trained on other domains to agricultural tasks, thereby saving time and resources. They also identify several challenges, such as the need for domain adaptation techniques and the potential for reduced accuracy when models are transferred across significantly different domains. The review concludes with a discussion of future research directions and the potential of emerging technologies to further advance precision agriculture. Hoque et al. (2024) incorporates meteorological data and pesticide information in order to predict crop yield. The authors perform experiments involving several ML models with gradient gradient-boosting model yielding the best results.

Lei et al. (2021) attempted to derive five vegetation indices from the multispectral UAV-acquired data and high-resolution UAV remote sensing images; this included the leaf chlorophyll index, the green normalized difference vegetation index, the normalized difference red-edge index, the optimal soil adjusted vegetation index, and the normalized difference vegetation index. To achieve this, five distinct algorithms are used on the data. The test set classification accuracy obtained from these classification algorithms through experiments resulted in 86.57% using BPNN.

Sharma et al. (2022) suggested plant disease diagnosis and image classification concerning rice and potato plants. They developed a CNN model for classifying diseases in rice and potato plant leaves. It can identify diseases including brown spot, blast, bacterial blight, and tungro in rice leaves, and it can categorize photos of potato leaves into three groups: illnesses of early and late blight, and healthy leaves. The proposed CNN model learned the hidden patterns from raw images to classify rice images with an accuracy of 99.58% and potato leaves with 97.66%. Shrivastava et al. (2019) presented an image-based machine-learning approach for the detection and classification of plant diseases, focusing on rice plant (Oryza sativa) diseases. Images of symptoms in leaves and stems due to those diseases were captured from rice fields. They extracted features through AlexNet and performed the classification through SVM. The result showed an Accuracy score of 91.37% the highest for their proposed system.

Plant diseases were recognized by Kaushik et al. (2022) for identifying diseases and preventing economic damage to farmers. The authors developed a DL system comprising the faster region-based CNN, region-based CNN fully, and R-CNN known as SSD for image recognition, and finally, the single-shot multi-box detector. The proposed technique copes with complex scenarios and efficiently identifies various diseases since they have managed to come up with a maximum accuracy of 94.6%. Xu et al. (2022); Mputu et al. (2024) proposes a new way to sort and grade the quality of tomatoes. This method is designed for feature extraction by pre-trained CNNs, belonging to a hybrid model because it combines the basic framework of the ML algorithm for classification. With Inception-V3 serving as the feature extractor, the CNN-SVM achieved the best performance suggested, accepting ripe, unripe, and rejecting tomatoes with an accuracy of 97.50% for the binary classification. Texture classification and analysis utilize LBP. LBP views the image in a localized way by looking at the relationships of the target pixel with its neighbors. It boosts accuracy, especially in small sizes of datasets and for the diversity of growth conditions. A summary of the above-discussed literature is given in **Table 1**.

**Table 1:** Summary of previous research works.

Ref	Classifiers	Dataset	Performance
Sachdeva et al. (2021)	Optimized CNN	Plant Village	98.9%
Zeng et al. (2021)	MobileNetV2	Plant Village	99.55%
Devi and Amarendra (2021)	LR, Linear SVM, HGB, NB, RBF-SVM, DT,	Plant Village	89.11% HGB
Mohanty et al. (2016)	GoogleNet, AlexNet	Plant Village	GoogleNet 99.34%
Wang et al. (2020)	KMSVM, KMSEG, Kmean, SVM, 2 class kmean,	Remote sensing Selfcollect UAV dataset	88.5% KMSEG
Lei et al. (2021)	DT, NB, SVM, k-NN, BPNN	Remote sensing UAV multispectral dataset	86.57% BPNN
Sharma et al. (2022)	CNN, SVM, KNN, DT, RF	rice and potato dataset	CNN: 99.58% on rice CNN: 97.66% on potato
Shrivastava et al. (2019)	AlexNet for extraction of features and SVM for classification	Self-collected rice dataset	91.37%
Kaushik et al. (2022)	Optimized CNN	Plant Village	94.6%
Mputu et al. (2024)	CNN-RF, CNN-SVM, CNN-kNN	Self-made	97.50% CNN-SVM

## 3 Materials and methods

**Figure 1:**
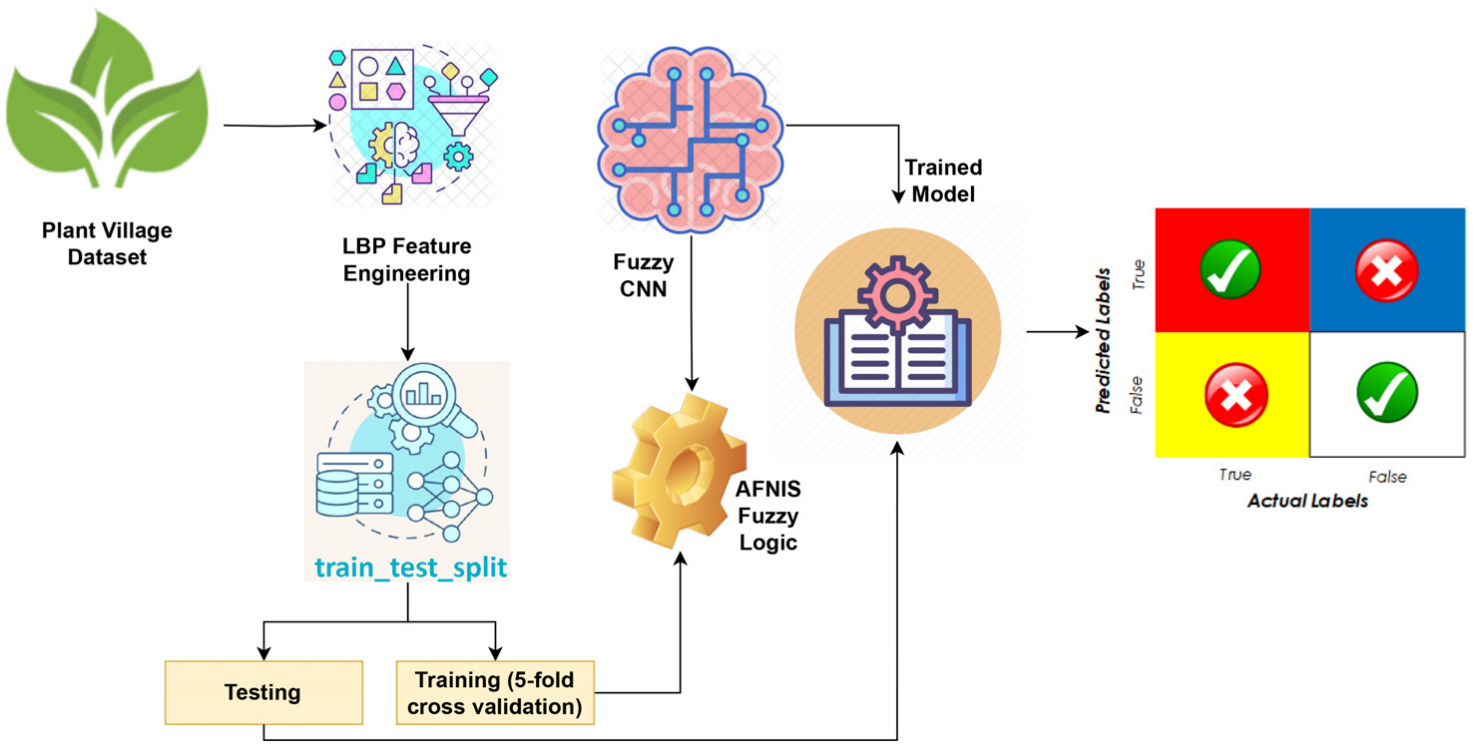
Proposed methodology diagram of Fuzzy-CNN model.

This section discusses ML and DL learning models along with the dataset utilized for the detection of leaf disease in plants. This section also describes about parameters used to assess the models’ performance and the suggested technique. The proposed methodology adopted in this study is shown in **Figure 1**.

### 3.1 Datasets description

The plant leaf disease dataset was taken from Kaggle, one of the top resources for research datasets. This specific dataset contains 2,475 images that are targeted for the pepper bell Tejaswi (2020). The actual labels are the images of leaves infected by bacteria and the healthy ones; they are two in number. This dataset comprises 1,478 images of healthy leaves and 979 images of infected leaves. The direct access link for the dataset is https://www.kaggle.com/datasets/arjuntejaswi/plant-village.

### 3.2 Local binary patterns

The LBP is a rather extensively used area in computer vision for texture analysis and classification.

LBP does not try to process the complete image but instead focuses on those areas that are localized by examining neighboring relationships between pixels Rosdi et al. (2011); Sun et al. (2023). Every pixel in an image considers a different set of neighborhoods. Comparisons within this testimonials scheme in the surrounding pixels are done concerning their intensity levels with the considered pixel and a central pixel. If the intensity in the neighboring pixel intensity is superior or equal to that in the central pixel, then it assigns one since it can be considered as a contrast in intensities. Therefore, for any region, a binary pattern is formed in which a value of zero is said to be the case if the intensity of the surrounding pixel is lower than that of the core pixel. Consequently, texture information from a region is contained in the decimal number that results from converting the binary pattern. Consequently, the LBP values’ histogram offers a simple textural representation of the information in a picture.

LBP offers substantial advantages for numerous applications, comprising, among many other things, object identification, face recognition, and texture classification. The robustness against changes in illumination enhances its effectiveness in scenarios where the light may vary. It has been further extended and modified in various ways, such as Uniform LBP, Rotation-Invariant LBP, and variations for multi-scale and multi-block LBP. It is easily understood by anyone that, being a texture descriptor, LBP is robust, whereas its performance is dependent on the application and dataset it uses. Also, pre-processing is carried out by normalizing the data and ensuring all images are converted to a standardized size of 230 *×* 230 pixels.

### 3.3 ML and DL learning models

ML is notable for supporting improvement in the accuracy and efficiency of disease diagnosis. There are various ML techniques, the most common being the Python package of Scikit-learn, which is highly used, open-source, and supported by a vast number of its users. We run this study with a variety of benchmark classifiers, including LR, RF, SVM, ETC, VGG19, EfficientNetB4, InceptionV3, and MobileNet, ResNet, in addition to the proposed ANFISFuzzyCNN. The following values are adopted for these models.

#### 3.3.1 Logistic-regression

In the realm of ML applications for plant leaf disease detection, LR serves as a valuable tool due to its simplicity, interpretability, and effectiveness in binary classification tasks Kleinbaum et al. (2002). Unlike its name implies, LR is adept at predicting binary outcomes by modeling the probability of an event (such as leaf disease presence) based on input features extracted from leaf images. This technique comes in very handy in analyzing different visual characteristics of leaves, such as color, texture, and shape, which can indicate the presence of disease. LR works by fitting a logistic function to the input features, mapping them linearly to output probabilities between 0 and 1. This allows for ease in interpretability of the coefficients, one gets an idea of how each feature contributes to the likelihood of disease while improving computational efficiency and scaling LR, as often large datasets are encountered in agricultural studies. Moreover, to guarantee robust performance on unseen data, LR can be regularized to prevent overfitting and, hence improve its generalization. This limits the power in the capturing of complex, nonlinear patterns in data as opposed to more sophisticated models. At the same time, the practical advantages of LR make it quite suitable for preliminary exploration into plant disease detection.

#### 3.3.2 Random forest

The RF model is a tremendous all-powerful tool used to detect plant leaf diseases using ML Breiman (2001). In general, the working principle of the RF algorithm is the aggregation of multiple decision trees built during the training process, which predicts the model’s output to improve accuracy and reliability. RF does an excellent job handling complex interactions between high-dimensional features extracted from an image of the leaf, including color, texture, and shape descriptors. Ensemble methods used by itself help to reduce overfitting. It also provides insight into feature importance, which is handy in knowing the critical markers through which the disease can be detected. Despite being a bit sensitive to the tuning of its various parameters, the capability of RF to capture complex patterns makes it very useful for applied agricultural research in enhancing crop health and management against diseases.

#### 3.3.3 Support vector machines

SVM is another activity of the more influential supervised learning models for classifying plant leaf diseases Genitha et al. (2019). SVM essentially works by finding the ideal feature space hyperplane that maximally separates the different classes. The concept not only made binary classification very effective but also easily placed into multi-class cases with methods such as one-vs-rest or one-vs-one classification. One of the main advantages of kernel functions in SVMs is that they make the technique very effective in nonlinear relationships between features and classes. Thus, due to the possibility of processing high-dimensional spaces of features, together with complex nonlinear relationships, SVMs are much preferred for the robust and accurate classification of plant diseases based on diverse features derived from images.

#### 3.3.4 Extra trees classifier

ETC is a meta estimator that considers the problem as a whole and, during training, creates many decision trees before presenting the mean forecast in the case of regression or the class mode in the case of classification Geurts et al. (2006). It is similar to RF but differs in how it selects splits at each node. ETC randomly selects splits, rather than searching for the best split like in traditional decision trees or RF, which can lead to faster training times. This approach can sometimes improve performance by introducing more randomness into the model, though it may also increase variance. ETC is useful in scenarios where computational efficiency is critical or where a higher level of randomness in model predictions is desired to prevent overfitting.

#### 3.3.5 Visual geometry group

VGG19 is a CNN architecture that achieves excellent performance for several image-processing assignments Younis et al. (2022). It has been developed by the Visual Geometry Group of Oxford University and contains 19 layers: 3 fully connected and 16 convolutional. VGG19 has an architectural design that stacks multiple layers of the pattern of convolution sizes with a stride of 3 *×* 3 before every application of this construct, with max pooling applied using a window of size 2 *×* 2 and stride 2. Such a structure helps VGG19 to capture the progressively complex features of an image. The cross-entropy loss criterion and optimization, which responds to iterations using stochastic gradient descent, comprise up’s general training setting of VGG19. VGG19 can thus be very easily interpreted and directly transferred to learning as well, given its absolute simplicity and uniform architecture. Correspondingly, the depth of the VGG19 architecture is the primary determinant of computational and memory consumption.

#### 3.3.6 EfficientNetB4

CNN-based EfficientNetB4 architecture is well-known for its effectiveness and exceptional performance in image recognition applications Wang et al. (2021). It belongs to the EfficientNet family, which systematically scales up the model’s depth, width, and resolution to achieve better accuracy without significantly increasing computational cost. In particular, EfficientNetB4 finds a balance between model accuracy and size, which makes it appropriate for a range of applications, including plant leaf disease detection using ML. By leveraging compound scaling and efficient building blocks, EfficientNetB4 optimizes both parameter efficiency and computational efficiency, keeping it competitively performant and well-suited for deployment on devices with limited resources in complex visual recognition tasks.

#### 3.3.7 InceptionV3

InceptionV3 is a frequently applied model in the CNNs for problems connected with image recognition. After being trained on the ImageNet dataset, it offers nearly all benchmarks with respectable accuracy Mujahid et al. (2022). Because of the several parallel layers of convolutional, pooling, and activation functions in its design, as well as inception modules, the network can learn diverse feature maps at varying sizes. Additionally, factorized 1 *×* 1 convolution and batch normalization have been included to minimize parameter sizes and improve training effectiveness. Besides being deep and computationally expensive, InceptionV3 is designed to be adaptable to other tasks and datasets; hence, It is appropriate for learning transfers. However, training and deployment can require a lot of memory and be computationally demanding.

#### 3.3.8 MobileNet

MobileNet is a unique design of CNN created for two primary reasons. First, for mobile phones, which have little processing power, and second, for embedded devices Ahsan et al. (2021). This balanced between the size of an accurate model and the accuracy. Depthwise Convolution applies a single filter to each input channel; this is the primary innovation of MobileNet. Depthwise Separable Convolutions essentially split standard convolutions into pointwise and depthwise convolutions. Then, pointwise convolution applies a 1 *×* 1 convolution to combine the results of the depthwise convolution. This separation greatly reduces computation and saves a great deal in model size compared to traditional convolution. For this reason alone, MobileNet is efficient in architecture with reasonable accuracy. There are several versions of MobileNet, MobileNetV1, MobileNetV2, and MobileNetV3, each of which provides a different set of improvements and optimizations over the previous one. These make the architecture efficient with much better performance. MobileNet has become popular on mobile and embedded devices in many computer vision applications, Examples include object identification, image categorization, and semantic segmentation.

#### 3.3.9 ResNet

ResNet, or Residual Network, is among the pioneering deep neural network architectures that revolutionized the scene within the domain of computer vision Fulton et al. (2019). The Microsoft Research team proposed residual learning to make the training of intense networks easier by residual connections through skip connections or shortcuts bypassing a few layers. These classes of shortcuts allow the gradient to flow more directly in backpropagation, and this alleviates the vanishing gradient problem, which enables models in ResNet to train efficiently with hundreds of layers. With such depth, ResNet can learn intricate features and patterns in data, hence providing state-of-the-art performance on a wide range of recognition tasks, including plant leaf disease classification. The relatively simple architectural backbone of ResNet and its effectiveness in learning complex representations have been the bedrock of DL research and applications.

### 3.4 Proposed approach ANFISFuzzyCNN for plant leaf disease detection

In this paper, ANFIS-Fuzzy-CNN represents a hybrid model proposed for the effective classification of plant diseases. This approach combines adaptive learning capabilities through fuzzy neural networks with the DL power of CNNs to enhance accuracy and robustness in agriculture-related disease classification systems. The complete details of the hyperparameters of the proposed model are shown in **Table 2**. The pseudo-algorithm of the proposed model is shared in **Algorithm 1**.

**Table 2:** Hyperparameter for the proposed model “ANFIS Fuzzy CNN”.

Hyperparameter	Value	Description
Input Shape	(224, 224, 3)	Size of the input image data (width, height, channels).
Convolutional Layers	3 layers [32, 64, 128 filters]	The number of convolutional layers used. The number of filters per layer.
Kernel Size	3x3	Size of the kernels used in convolutional layers.
Activation Function	ReLU	Activation function applied after each convolutional layer.
Pooling Layers	2 layers	Number of pooling layers used.
Pooling Type	Max Pooling	Type of pooling used in pooling layers.
Pooling Size	2x2	Size of the pooling window.
Fuzzy Layer Type	Gaussian	Type of fuzzy layer used for feature extraction.
Dropout Rate	0.5	Rate of Dropout applied after fully connected layers.
Batch Size	32	Number of samples per gradient update.
Number of Epochs	50	Total number of iterations over the entire dataset.
Optimizer	Adam	Optimization algorithm used for training the model.
Learning Rate	0.001	Initial learning rate for the optimizer.
Loss Function	Categorical Crossentropy	Loss function used for optimization.

Neuro-fuzzy is another hybrid approach targeting the combination of Fuzzy logic and neural networks to solve most real-world problems efficiently Jang (1993). In a sense, the combination is intended to complement the deficiencies of basic models: neural networks are excellent in pattern recognition but usually remain ‘black boxes’ concerning explanation of their decisions; on the other hand, fuzzy logic systems support imprecise reasoning but are very good at explaining their decisions [11]. In this respect, a famous Neuro-Fuzzy architecture is proposed, i.e., the ANFIS. The ANFIS part of the model uses fuzzy logic to handle the existing uncertainty and imprecision in plant disease classification. In this line, fuzzy logic can provide a means for representing vague concepts or linguistic variables, especially in agricultural domains where diseases’ symptoms are subjective or relative, depending on appearance and severity. ANFIS dynamically adapts the fuzzy rules and membership functions concerning the input data to classify optimally. It is this adaptability that is core to handling a diverse group of plant diseases and variations in environmental conditions.

Due to their capability of automatically identifying hierarchical features from unprocessed input data, in this case, images of plant leaves affected by diseases, CNNs have been of great importance in ANFIS-Fuzzy-CNN. As such, they are very well suited for image-based tasks like disease detection since meaningful spatial and temporal patterns could be extracted using multiple layers of convolutions with subsequent pooling operations. These convolution layers allow the model to capture minute details and textures indicative of different plant diseases, thus improving classification accuracy. ANFIS integration into CNN in the ANFIS-Fuzzy-CNN model is synergistic. In ANFIS, it introduces a fuzzy inference mechanism working on the outputs from CNN. This work refines the classification decisions of the CNN with fuzzy logic rules that consider uncertainty and context-specific knowledge about plant diseases. The ANFIS-Fuzzy-CNN model first stepped with a convolutional network that extracted features from input images which went through a few layers of convolutions and pooling Sabrol and Kumar (2020). These features are then processed by a fuzzy inference system where the extracted features from the smart camera are analyzed with the help of fuzzy rules that deal with uncertainties and ambiguity which are mostly inherent in real-world scenarios. One of the most advantageous features of the system is the ability to adjust its parameters that are trained to ensure better solutions are gotten especially for compound classifications.

Algorithm 1 **ANFIS Fuzzy CNN for plant leaves classification.**

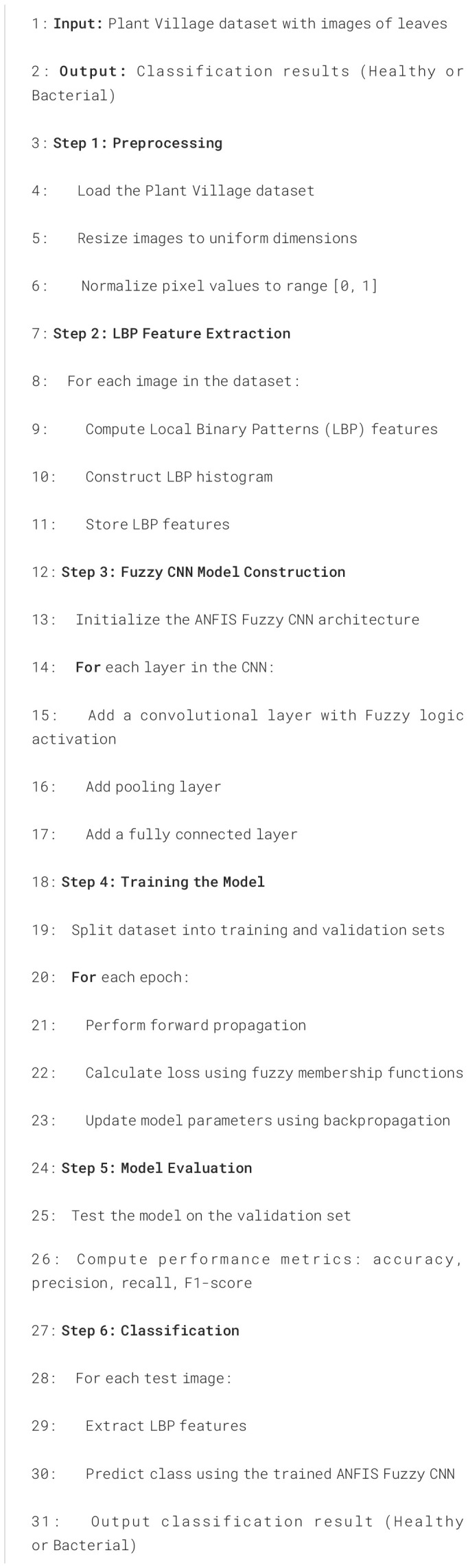

Compared with traditional ML methods for plant disease classification, the ANFIS-Fuzzy-CNN model has the following advantages: First, it can handle complicated and nonlinear relationships between input features (e.g., symptoms of diseases) and output classes (certain diseases). Second, it improves the robustness of classification by generalizing the strengths of fuzzy inference systems in handling uncertainty with the capability of CNNs in understanding hierarchical representations from raw data. The equation below illustrates a simplified form of how ANFIS integrates with CNN for disease classification:


(1) $${\text{Output}}_{label}=\text{ANFIS}\left(\text{CNN}\left({\text{Input}}_{image}\right)\right)$$

where $(\text{CNN}\left({\text{Input}}_{image}\right)$ represents the feature extraction and classification stages performed by the CNN, while ANFIS CNN refines the output label based on fuzzy inference rules.

### 3.5 Evaluation metrics

This study utilizes different evaluation criteria, including accuracy, F1-score, precision, and recall. These assessment metrics help determine whether ML and DL models are efficient.

An overall assessment of the model’s prediction is provided by accuracy. This statistic calculates the proportion of accurately predicted instances to all instances in a dataset. It can be determined by the formula.


(2) $$Accuracy=\frac{\sum_{i=1}^{C}{\text{TP}}_{i}}{\sum_{i=1}^{C}{\text{N}}_{i}}$$

Precision is a measure employed to ascertain the ratio gotten from the correct predicted positive instances divided by the total that was predicted positive. In layperson’s terms, this helps to minimize the number of false positives, clearly showing the distinction of the model towards positive instances. Precision can be given as:


(3) $$Precision_{i}=\frac{{\text{TP}}_{i}}{{\text{TP}}_{i}+{\text{FP}}_{i}}$$

Recall, otherwise called sensitivity, at the same time is the proportion of the number of valid positive instances predicted by the number of genuine positive cases in the dataset’s class. In a more general way, it shows how well the model can detect real positive cases. The formula for the recall is:


(4) $$Recall_{i}=\frac{{\text{TP}}_{i}}{{\text{TP}}_{i}+{\text{FN}}_{i}}$$

The F1 score is the harmonic mean of precision and recall and thus provides a measure that balances both metrics about the general behavior of the model. It is calculated with the formula:


(5) $$F1-Score=2\cdot \frac{{\text{Precision}}_{i}\cdot {\text{Recall}}_{i}}{{\text{Precision}}_{i}+{\text{Recall}}_{i}}$$

### 3.6 Experimental configuration

This study utilized TensorFlow and Keras libraries, both open-source, to create various ML and DL models, some of which were pre-trained. DL methods were utilized on a collection of plant leaf pictures with Python on the Anaconda software. To conduct experiments on datasets using advanced DL models such as ANFIS Fuzzy CNN, this research utilized a system with an Intel Core i9-11900K processor, which features 8 cores and 16 threads, operating at a base frequency of 3.5 GHz. This processor provides the necessary computational power for handling complex data preprocessing and model training. The system is equipped with a high-performance GPU, NVIDIA GeForce RTX 3080, which offers 10 GB of GDDR6X VRAM. This GPU accelerates the training of DL models by leveraging parallel processing capabilities. To support the high computational demands, the system had 64 GB of DDR4-3600 RAM, ensuring smooth operation and efficient handling of large datasets. To identify bacteria in images of plant leaves, it is suggested to use the ANFIS-Fuzzy-CNN DL model in conjunction with LBP. Different scientific methodologies will be used to assess how effective and important the proposed approach is. This research focuses on categorizing diseases found on pepper bell plant leaves utilizing a dataset containing 2,475 images. To accomplish this goal, scientists employed the ANFIS-Fuzzy-CNN model along with LBP.

## 4 Results and discussion

Four ML models were used on the original dataset to classify pepper bell plant leaf diseases without taking into account LBP features. **Table 3** presents the experimental results for these ML models.

**Table 3:** ML models results using original dataset.

Model	Accuracy	Precision	Recall	F1-Score
RF	0.7548	0.8155	0.8234	0.8207
LR	0.7629	0.8367	0.8449	0.8419
ETC	0.8034	0.8569	0.8569	0.8569
SVM	0.7249	0.7939	0.8046	0.7969

**Table 3** shows the resultant performance metrics for various ML models using the original dataset. Every model is assessed using Accuracy, F1-Score, Precision, and Recall. They are Random Forest. The accuracy ranged from 72.49% using SVM to 80.34% using ETC—Late, thus telling how often each model has predicted outcomes correctly. Precision ranges from 79.39% in the case of SVM to 85.69% for ETC. It indicates the proportion of all expected positives among those predicted as positive. Recall values range from 80.46% (SVM) to 85.69% (ETC), reflecting how well each model identifies actual positive instances. The F1-Scores range from 79.69% (SVM) to 85.69% (ETC), which combines Precision and Recall into a single metric, illustrating the overall effectiveness of each model in making accurate predictions.

### 4.1 TL models results using original dataset

TL models showed encouraging outcomes for the image data. This study utilizes five TL models along with the proposed ANFIS-Fuzzy-CNN. **Table 4** shows the outcomes of TL classifiers and the proposed system using the original dataset.

**Table 4:** TL models results without LBP.

Models	Accuracy	Precision	Recall	F1 score
MobileNet	0.7969	0.8134	0.8391	0.8248
VGG19	0.7999	0.8263	0.8367	0.8219
ResNet	0.7734	0.8025	0.8319	0.8267
EfficientNet B4	0.8265	0.8769	0.8932	0.8855
**ANFIS-Fuzzy-CNN**	**0.8478**	**0.8959**	**0.9045**	**0.8953**

Bold values indicate the proposed model results.

**Table 4** the performance of various TL models without using LBP. Each model—MobileNet, VGG19, ResNet, EfficientNet B4, and ANFIS-Fuzzy-CNN—is evaluated based on Accuracy, Precision, Recall, and F1 score. Accuracy ranges from 77.34% for ResNet to 84.78% for ANFIS-Fuzzy-CNN, indicating overall prediction correctness. Precision values vary from 80.25% (ResNet) to 89.59% (ANFIS-Fuzzy-CNN), showing the proportion of correctly predicted positive cases out of all predicted positive cases. Recall ranges from 83.19% (ResNet) to 90.45% (ANFIS-Fuzzy-CNN), indicating how well each model identifies actual positive instances. F1 scores range from 82.19% (VGG19) to 88.55% (EfficientNet B4), providing a combined measure of Precision and Recall, reflecting each model’s overall performance in making accurate predictions.

### 4.2 Performance of ML models using LBP features

This research uses the LBP model for the textual descriptor. In the dataset, ML models are used after carrying out the Local Binary Pattern. **Table 5** shows how ML models using Local Binary Patterns perform.

**Table 5:** Results of ML models with LBP.

Models	Accuracy	Precision	Recall	F1 score
RF	0.8938	0.9137	0.8234	0.8207
LR	0.8749	0.8367	0.8449	0.8419
ETC	0.9182	0.8569	0.8569	0.8569
SVM	0.9222	0.7939	0.8046	0.7969

The table shows the performance of various TL models without using LBP. Each model, MobileNet, VGG19, ResNet, EfficientNet B4, and ANFIS-Fuzzy-CNN, is evaluated based on accuracy, precision, recall, and F1 score. Accuracy ranges from 77.34% for ResNet to 84.78% for ANFIS-Fuzzy-CNN, indicating overall prediction correctness. Precision values vary from 80.25% (ResNet) to 89.59% (ANFIS-Fuzzy-CNN), showing the proportion of correctly predicted positive cases out of all predicted positive cases. Recall ranges from 83.19% (ResNet) to 90.45% (ANFIS-Fuzzy-CNN), indicating how well each model identifies actual positive instances. F1 scores range from 82.19% (VGG19) to 88.55% (EfficientNet B4), providing a combined measure of precision and recall, reflecting each model’s overall performance in making accurate predictions.

### 4.3 Performance of TL models using local binary pattern

**Figure 2:**
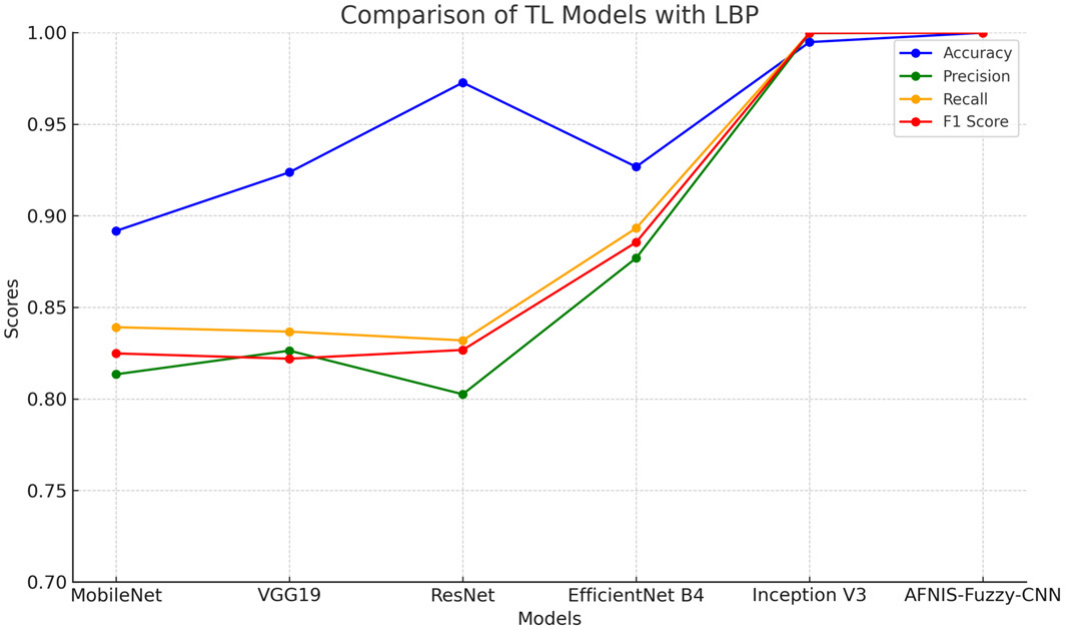
Comparison line-graph of TL models using LBP features.

The data after applying LBP are fed into TL models. **Table 6** and **Figure 2** show the performance of TL models on LBP data.

**Table 6:** Results of TL models with LBP.

Models	Accuracy	Precision	Recall	F1 score
MobileNet	0.8917	0.8134	0.8391	0.8248
VGG19	0.9237	0.8263	0.8367	0.8219
ResNet	0.9727	0.8025	0.8319	0.8267
EfficientNet B4	0.9267	0.8769	0.8932	0.8855
Inception V3	0.9848	0.9997	0.9999	0.9998
**ANFIS-Fuzzy-CNN**	**0.9999**	**0.9999**	**0.9999**	**0.9999**

Bold values indicate the proposed model results.

**Table 6** compares the performance of various TL models enhanced with LBP. In this respect, each of the models, including MobileNet, VGG19, ResNet, EfficientNet B4, and Inception V3, together with ANFIS-Fuzzy-CNN, are plugged into criteria such as the F1 score, accuracy, precision, and recall. The accuracy ranges from 89.17% for MobileNet to 98-99% for ANFIS-Fuzzy-CNN and Inception V3, meaning almost all the predictions are generally correct. The values obtained for precision are 80.25% for ResNet and 99.99% for both ANFIS-Fuzzy-CNN and Inception V3, respectively. Recall ranges from 83.19% in the case of ResNet to as high as 99.99% in ANFIS-Fuzzy-CNN and Inception V3, which means that each model recalled actual positive instances of a class this much. F1 ranges from 82.19% in VGG19 to 99.98% in Inception V3. It does exactly what an F1 score should do, combining precision and recall into a combined measure reflecting how well each model makes accurate predictions with the addition of LBP.

### 4.4 Discussion

The proposed ANFIS Fuzzy CNN model excels in plant leaf disease detection due to several key innovations. It integrates an ANFIS which allows the model to dynamically adjust to variations in plant leaf images, thereby extracting more relevant and distinctive features. The incorporation of fuzzy logic enhances the model’s ability to handle uncertainty and variability in agricultural data, making it more resilient to noise and variations in image quality. The model’s CNN structure is adept at capturing intricate patterns and textures, which are crucial for accurate disease classification. Furthermore, the proposed model benefits from prior knowledge while adapting to the specific nuances of plant leaf disease detection, reducing training time. The model’s performance is validated using various metrics, such as accuracy, F1-score, precision, and recall providing a holistic view of its effectiveness. Rigorous cross-validation and benchmarking against state-of-the-art models and datasets demonstrate the model’s consistency and superior performance, justifying its robustness and efficacy in precision agriculture applications. We performed an ablation study and the results are given in **Table 7**. Results show the impact of each layer and component on the performance of the proposed ANFIS Fuzzy CNN model.

**Table 7:** Mean accuracy of ANFIS Fuzzy CNN model with respect to various parameters.

Parameter	Mean Accuracy (%)
Input Size 128x128	95.75
Input Size 256x256	97.50
Fuzzy Layers 1	95.67
Fuzzy Layers 2	97.67
Fuzzy Layers 3	94.33
Regularization 0.01	98.25
Regularization 0.1	96.67
Regularization 1.0	94.00
Learning Rate 0.001	97.75
Learning Rate 0.01	97.00
Learning Rate 0.1	93.00
Batch Size 16	95.00
Batch Size 32	97.00
Batch Size 64	98.50

### 4.5 Results of k-fold cross-validation

In this study, k-fold cross-validation has been implemented to enhance the performance analysis of the proposed method. The computed results of 5-fold cross-validation are shown in **Table 8**, indicating that the technique proposed is effective concerning various metrics. The standard deviation is found to be very low, indicating better consistency in performance across folds. These results add to the confidence level in the degree of reliability and credibility of the proposed technique.

**Table 8:** Cross-validation results.

Folds	Accuracy	Precision	Recall	F1-Score
First-Fold	0.9997	0.9973	0.9985	0.9990
Second-Fold	0.9994	0.9964	0.9995	0.9994
Third-Fold	0.9993	0.9996	0.9996	0.9996
Fourth-Fold	0.9999	0.9993	0.9997	0.9993
Fifth-Fold	0.9999	0.9995	0.9999	0.9999
**AVG**	**0.9996**	**0.9984**	**0.9994**	**0.9994**

Bold values indicate the proposed model results.

**Figure 3:**
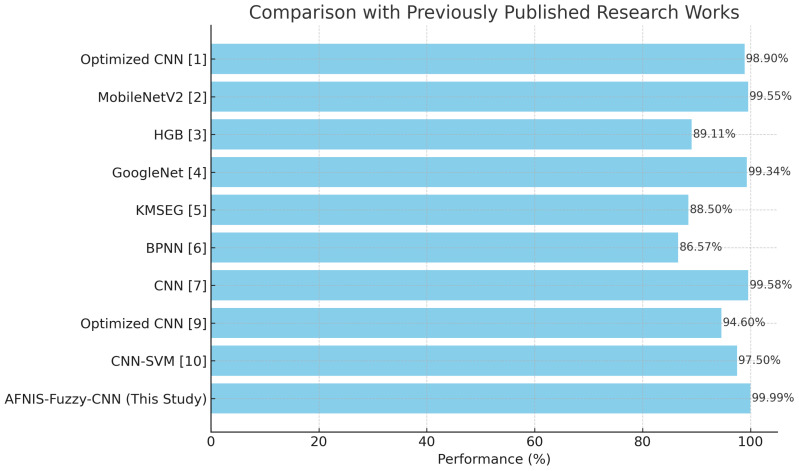
Comparison bar-graph with previously published research works.

**Table 8** and **Figure 3** show the summary of performance metrics using cross-validation with LBP. Again, in this case, there is also available for every fold; first-fold to fifth-fold, metrics such as precision, F1 score, accuracy, and recall. The accuracy in each fold comes very close: 99.93% to 99.99%, hence very accurate. The precision values are always very high, in the range of 99.64% to 99.96%, indicating that out of all positives predicted, the models could get most of the positive cases right. Recall values are also exceptionally high, ranging from 99.85% to 99.99%, thus indicating how well the models can capture positive instances. Another combined measure for precision and recall is the F1 score, which ranges from 99.30% to 99.99%. This again proves the overall sound performance in both precision and recall metrics. The final AVG metrics across folds underpin the excellent quality of the models in terms of average accuracy, precision, recall, and F1-score with corresponding values of 99.96%, 99.84%, 99.94%, and 99.94%. These results prove that with k-fold cross-validation and LBP, high accuracy and reliability were obtained in the models trained for outcome prediction.

The proposed ANFIS Fuzzy CNN model offers a more adaptive and context-aware approach compared to the deep convolutional neural networks (CNNs) employed in the referenced research. While studies like Sachdeva et al. (2021) and Zeng et al. (2021) leverage CNNs and transfer learning for plant disease detection, their models lack the fuzzy logic integration that enhances spatial coherence in ANFIS. Similarly, the HistGradientBoosting model used by Devi and Amarendra (2021) combines traditional feature extraction methods like HOG and LBP, but does not achieve the same adaptability in complex environments as ANFIS. Furthermore, while Mohanty et al. (2016) and Wang et al. (2020) employ UAVs and remote sensing for plant disease detection, ANFIS outperforms these methods in terms of accuracy and robustness, especially in dynamic scenarios. The key advantage of ANFIS is its ability to incorporate fuzzy logic for more precise feature extraction, setting it apart from traditional deep CNNs and transfer learning methods.

**Table 9** captures a performance comparison between the proposed model and SOTA. It shows ANFIS-Fuzzy-CNN performing better than other SOTA models in terms of accuracy, as depicted in the **Table 9**. The model’s comparison is illustrated in **Table 10**.

**Table 9:** Comparison with previously published research works.

Reference	Classifiers	Dataset	Accuracy	Limitations
Sachdeva et al. (2021)	Optimized CNN	Plant Village	98.9%	no ANFIS feature capturing, no cross-validation, no pre-processing
Zeng et al. (2021)	MobileNetV2	Plant Village	99.55%	no ANFIS feature capturing, no cross-validation, no pre-processing
Devi and Amarendra (2021)	HGB	Plant Village	89.11%	no ANFIS feature capturing, no cross-validation
Mohanty et al. (2016)	GoogleNet	Plant Village	99.34%	no ANFIS feature capturing, no cross-validation
Kaushik et al. (2022)	Optimized CNN	Plant Village	94.60%	no ANFIS feature capturing, no cross-validation
**This study**	**ANFIS-Fuzzy-CNN**	**Plant Village**	**99.99%**	all limitations addressed

Bold values indicate the proposed model results.

**Table 10:** Comparison of ANFIS Fuzzy CNN with other models.

Model	Complexity	Parameter Count	Accuracy	Suitability	Key Advantages
**ANFIS Fuzzy CNN**	High (adaptive sampling + fuzzy logic)	Medium to High	High (for spatial coherence)	Precision tasks (e.g., medical imaging, leaves fine details, image segmentation)	Adaptive and context-aware feature extraction
**Deep CNN**	Medium (straightforward architecture)	Medium to High	High for general tasks	General purpose tasks like image classification	Simplicity and broad application
**MobileNetV2**	Low (optimized for efficiency)	Low	Medium to High	Mobile and embedded applications	Computational efficiency and low resource usage
**HGB CNN**	Very High (CNN + gradient boosting)	Very High	High	Complex tasks (e.g., multi-label classification)	Combines CNN and boosting for better decisions
**GoogleNet**	Medium to High (inception modules)	Medium	High	Largescale tasks, multiscale feature extraction	Efficient multiscale feature extraction

### 4.6 Real-world applications of proposed approach

Plant diseases are a real threat to agriculture and their robust detection is challenging. Devising a framework that can perform timely and accurate disease detection holds great promise for farmers. The proposed approach shows superb results and can be used for real-world disease detection thereby helping better and timely countermeasures to avoid crop losses. It can help farmers get better yield thereby improving their earnings and contributing to the economy. The proposed approach provides robust results and can be utilized for disease detection at an early stage of disease which can be very influential to reduce disease losses. For deployment, the system can be integrated into edge devices like mobile phones or dedicated cameras for field use, and utilize cloud-based systems for extensive processing and storage. In addition, it can be incorporated into smart farming where the real-time data can be fed to the model for disease detection. The output of the framework can be linked to a smartphone app for real-time updates to the farmers improve their decision making.

### 4.7 Limitations of proposed ANFIS Fuzzy CNN model

Despite the promising performance of the proposed ANFIS Fuzzy CNN model in detecting plant leaf diseases, some limitations must be considered. Firstly, the model’s reliance on large amounts of labeled data for training poses a significant challenge, as acquiring high-quality annotated images of various leaf diseases is time-consuming and labor-intensive. This can lead to issues of data imbalance, where some disease classes are underrepresented, potentially biasing the model’s predictions. Additionally, the computational complexity of the model, due to its fuzzy and convolutional components, may result in high training and inference times, making it less suitable for real-time applications in resource-constrained environments such as small farms.

## 5 Conclusion

Keeping in view the importance of timely disease detection, this study proposes a novel approach using leaf images. The proposed model for the pepper bell leaf disease detection utilizes ANFIS Fuzzy CNN and local binary pattern features. Without using the local binary pattern features, the achieved accuracy is 0.8478 while using the features demonstrates exceptional performance with a remarkable 99.99% in accuracy, precision, recall, and F1 score. The comparison with original image features yielded significantly lower results, highlighting the effectiveness of the proposed approach. Extensive experiments with various machine and DL models reaffirm the superiority of the proposed model. Additionally, cross-validation and comparisons with state-of-the-art techniques underscore its robustness and reliability. These findings emphasize the potential of the proposed method to revolutionize disease detection in agricultural practices, offering a highly accurate and efficient solution for early disease identification and management. The future work direction of this research work is the addition of explainable AI and Model Interpretability to give more insights into how these predictions are made. The second direction is the integration of a multi-modal fusion approach to make it a more reliable solution in the agriculture sector.

## Data Availability

Publicly available datasets were analyzed in this study. This data can be found here: https://www.kaggle.com/datasets/vipoooool/new-plant-diseases-dataset.
